# Constitutive equation of friction based on the subloading-surface concept

**DOI:** 10.1098/rspa.2016.0212

**Published:** 2016-07

**Authors:** Koichi Hashiguchi, Masami Ueno, Takuya Kuwayama, Noriyuki Suzuki, Shigeru Yonemura, Nobuo Yoshikawa

**Affiliations:** 1 MSC Software Ltd, Shinjuku First West 8F, 23-7 Nishishinjuku, 1-chome, Tokyo 160-0023, Japan; 2 Department of Agricultural Engineering, University of the Ryukyus, 1 Senbaru, Nishihara, Okinawa 903-0213, Japan; 3 Steel Research Laboratories, Nippon Steel and Sumitomo Metal Corporation, 20-1 Shintomi, Futtsu, Chiba 293-8511, Japan

**Keywords:** friction, overstress model, rate sensitivity, sliding, subloading-surface model

## Abstract

The subloading-friction model is capable of describing static friction, the smooth transition from static to kinetic friction and the recovery to static friction after sliding stops or sliding velocity decreases. This causes a negative rate sensitivity (i.e. a decrease in friction resistance with increasing sliding velocity). A generalized subloading-friction model is formulated in this article by incorporating the concept of overstress for viscoplastic sliding velocity into the subloading-friction model to describe not only negative rate sensitivity but also positive rate sensitivity (i.e. an increase in friction resistance with increasing sliding velocity) at a general sliding velocity ranging from quasi-static to impact sliding. The validity of the model is verified by numerical experiments and comparisons with test data obtained from friction tests using a lubricated steel specimen.

## 1. Introduction

The *rate-and-state friction model* (e.g. [1–4]), the basic idea of which is the dependence of the shear stress or friction coefficient on the rate of sliding and some state variables based on experimental data [5,6], has been widely used for the prediction of earthquake phenomena and occasionally for characterizing solid friction (e.g. [7]). An earthquake is a typical irreversible phenomenon which can be described appropriately by elasoplasticity but the rate-and-state friction model is not based on elastoplasticity; the latter is premised on (i) decomposition of the rate of deformation or sliding into the reversible, i.e. elastic part and the irreversible, i.e. plastic part, (ii) incorporation of the yield condition, and (iii) the potential flow rule of plastic strain rate or plastic sliding rate. Therefore, the rate-and-state friction model would be limited to one-dimensional sliding, as seen in earthquake faults in which the sliding direction does not vary markedly.

Constitutive equations of friction within the framework of elastoplasticity were formulated first as rigid-plasticity [8,9]. Subsequently, they were extended to elasto-perfect-plasticity [10–16]. Further, isotropic hardening was introduced [17,18]. However, these equations fall within the framework of conventional elastoplasticity, which assumes a friction-yield surface enclosing a purely elastic domain. Therefore, they are incapable of describing not only the accumulation of plastic sliding displacement during cyclic loading of contact stress but also the transition from static to kinetic friction and the recovery of static friction.

*Conventional elastoplasticity models* introduce the yield surface enclosing a purely elastic domain [19]. The models, therefore, have limitations: (i) the plastic (irreversible) strain rate due to the rate of stress inside the yield surface cannot be predicted and (ii) the smooth transition from the elastic to the plastic state is not described but an abrupt transition is described, and hence an accumulation of the plastic strain under cyclic loading of stress even at high-stress levels near the yield stress cannot be predicted. In addition, the models require some cumbersome procedures: (iii) yield judgement is required in the analyses and thus the determination of the yield point, i.e. the offset value (plastic strain) for yielding is required, which is accompanied by arbitrariness and cumbersomeness and (iv) an operation to pull back the stress to the yield surface is required when stress jumps out from the yield surface in numerical calculations.

All the above-mentioned limitations of conventional elastoplasticity models have been resolved by the *subloading*-*surface model* [20–23], as explained in the following.

The basic features of the formulation in the subloading-surface model are as follows. (i) The plastic strain rate develops continuously as the stress approaches the yield surface, which is renamed the *normal-yield surface*. (ii) The *subloading*-*surface* that passes through the stress and has a similar shape and orientation to the normal-yield surface, and the ratio of the size of the subloading-surface to that of the normal-yield surface, called the *normal-yield ratio*, which ranges from zero to unity, are introduced to describe the degree of approach of stress to the normal-yield surface. (iii) The evolution rule of the normal-yield ratio is formulated such that the ratio is attracted to unity in the plastic loading process. (iv) The plastic strain rate is formulated so as to develop continuously as the normal-yield ratio approaches unity. Then, the elastoplastic deformation behaviour is described in the subloading-surface model as follows. (i) A smooth elastic–plastic transition is described. (ii) Yield judgement is not required in the analyses and thus, needless to say, the determination of the offset value for yielding is not required. (iii) Stress is automatically attracted to the normal-yield surface during the plastic loading process, leading to high efficiency and accuracy in numerical calculations.

Consequently, the subloading-surface model is regarded as an appropriate constitutive model for a wide class of irreversible mechanical phenomena, as follows. (i) Monotonic and cyclic loading behaviours can be described realistically [23]. (The standard implementation of the subloading-surface model to the commercial finite-element method software Marc (MSC Software Ltd) will soon be available to Marc users.) (ii) The overstress model is modified to be applicable to the description of elastoplastic deformation at the general rate, ranging from quasi-static to impact loading [22]; the previous overstress model is not applicable to impact loading, as it results in an unrealistic prediction of only an elastic response with infinite strength. (iii) The damage model [24] and the phase transformation model [25] are modified to describe softening behaviour appropriately. (iv) The basis for constitutive modelling of the fatigue phenomenon induced under a low-stress amplitude is established [26]. (v) Exact finite strain elastoplasticity (hyperelastic-based plasticity) based on the multiplicative decomposition of the deformation gradient is formulated rigorously, as done by Hashiguchi & Yamakawa [27] for monotonic loading behaviour and by Hashiguchi [28] for general loading behaviour including cyclic loading behaviour. It is desirable to formulate a constitutive equation with a high generality and the universality, while any unconventional model, i.e. a cyclic plasticity model other than the subloading-surface model, has not been extended to multiplicative finite strain theory. (vi) The crystal plasticity analysis, in which slip analyses in numerous slip systems are required, is realized rationally with high efficiency because a yield judgement is not required and the stress is pulled back automatically to the yield surface [28,29]. Consequently, the subloading-surface model is valid for the description of irreversible mechanical behaviour in solids for rate-independent and rate-dependent behaviours ranging from the micro- to the macro-level for pressure-independent and pressure-dependent materials for finite deformation, combining both physical relevance and numerical efficiency.

We now concentrate our discussion on the friction phenomena in solids. With the exception of an isolated body floating in a vacuum, all bodies in the natural world are in contact with other bodies and thus subject to friction. The theoretical basis for the description of friction phenomena was established by the friction model based on the concept of the subloading surface, called the *subloading-friction model* [30,31]. The simplest formulation of the subloading-friction model also provides the basis for crystal plasticity analysis [28,29]. The subloading-friction model assumes that the plastic sliding velocity develops gradually as the contact stress (traction) approaches the friction-yield surface, based on the concept of the subloading surface [20,21,29]. It describes the friction resistance such that it first reaches a peak, i.e. static friction, then gradually decreases to a minimum, i.e. kinetic friction, and it recovers as sliding stops or sliding velocity decreases. The model is based on the fact that the friction coefficient decreases by plastic sliding, which breaks the adhesion between surface asperities on the contact surface, but it recovers with time, which causes the reconstruction of adhesion. Thus, *negative rate sensitivity* (i.e. a decrease in friction resistance with increasing sliding velocity) is described; such sensitivity is observed in dry friction without lubrication. By contrast, the *positive rate sensitivity* (i.e. an increase in the friction resistance with increasing sliding velocity) is observed in the sliding between lubricated surfaces or between soft solids, e.g. indium, Teflon and various polymers, which is often called the *fluid friction* or the *wet friction*.

Dry friction and fluid friction exhibit high and low friction resistances and large and small differences between static friction and kinetic friction, respectively. Furthermore, the stick–slip phenomenon is often induced by dry friction [32], in which friction resistance fluctuates upwards and downwards intensely and intermittently. It should be avoided in machine elements such as gears and bearings in order to induce smooth movement. By contrast, fluid friction with a positive rate sensitivity does not cause the stick–slip phenomenon, but its theoretical prediction is of importance for mechanical designs of lubricated machinery elements such as gears and bearings in which low friction resistance is desirable and of wheel tyres travelling on wet roads in which high friction resistance is required for high braking performance, and so forth.

This paper begins by giving a detailed physical interpretation of the subloading-friction model, which is then extended to the generalized subloading-friction model. The generalized model is capable of describing not only the negative rate sensitivity but also the positive rate sensitivity for an arbitrary sliding velocity ranging from quasi-static to impact sliding by introducing the concept of overstress [33,34] for the elasto-viscoplastic deformation behaviour. Numerical experiments for several settings of material parameters are presented to clarify the effects of the material parameters on friction resistance and the physical meanings of these parameters contained in the subloading-overstress friction model. Furthermore, friction tests using a lubricated steel specimen are performed to examine the validity of the model. These test results are reproduced in simulations using the subloading-overstress friction model. Consequently, it is verified that the subloading-overstress friction model is capable of describing friction phenomena with positive rate sensitivity in addition to negative rate sensitivity for an arbitrary sliding velocity ranging from quasi-static to impact sliding.

## 2. Physical interpretation and basic formulation of the subloading-friction model

The subloading-friction model [30,31] for negative rate sensitivity observed in dry friction is reviewed below. We add concise physical interpretations along with the basic mathematical formulation in preparation for the generalization of the model to describe not only negative but also positive rate sensitivities for an arbitrary sliding velocity.

### (a) Definitions of basic mechanical variables

The sliding velocity vector $\bar{\text{v}}$, which is defined as the relative velocity of the counter (slave) body to the main (master) body, is orthogonally decomposed into the normal sliding velocity vector ${\bar{\text{v}}}_{n}$ and the tangential sliding velocity vector ${\bar{\text{v}}}_{t}$ to the contact surface

2.1 $$\bar{\text{v}}={\bar{\text{v}}}_{n}+{\bar{\text{v}}}_{t}=-{\bar{v}}_{n}\text{n}+{\bar{v}}_{t}{\text{t}}_{v},$$

where

2.2 $${\bar{\text{v}}}_{n}=(\bar{\text{v}}•\text{n})\text{n}=(\text{n}⊗\text{n})\bar{\text{v}}=-{\bar{v}}_{n}\text{n},\;{\bar{\text{v}}}_{t}=\bar{\text{v}}-{\bar{\text{v}}}_{n}=(\text{I}-\text{n}⊗\text{n})\bar{\text{v}}={\bar{v}}_{t}{\text{t}}_{v}.$$

**I** is the second-order identity tensor possessing the components of Kronecker’s delta *δ*_*ij*_ (*δ*_*ij*_=1 for *i*=*j* and *δ*_*ij*_=0 for *i*≠*j*), **n** is the unit outward-normal vector of the main body, and

2.3 $${\bar{v}}_{n}\equiv -\text{n}•{\bar{\text{v}}}_{n}=-\text{n}•\bar{\text{v}},\;{\bar{v}}_{t}=∥{\bar{\text{v}}}_{t}∥,\;{\text{t}}_{v}\equiv \frac{{\bar{\text{v}}}_{t}}{∥{\bar{\text{v}}}_{t}∥}\;(\text{n}•{\text{t}}_{v}=0,∥{\text{t}}_{v}∥=1).$$

The minus sign is added to the definition of ${\bar{v}}_{n}$ so that friction increases as the counter body approaches the main body. The symbolic notations **u**⊗**vw**=**u**(**v**•**w**) and (**Tv**)_*i*_=*T*_*ir*_*v*_*r*_ with Einstein’s summation convention being used for arbitrary vectors **u**, **v**, **w** and arbitrary second-order tensor **T**, and ∥ ∥ denotes the magnitude (i.e. norm). An observed velocity of a material particle depends on the velocities of the observer and is thus not an objective quantity. On the other hand, the observed velocities of material particles of the main and the counter bodies on a contact surface are affected identically by the velocity of the observer, noting that these material particles possess the same position. Consequently, their relative velocity, i.e. the sliding velocity $\bar{\text{v}}$, is an objective quantity, which is independent of the observer.

Here, it is further assumed that $\bar{\text{v}}$ is additively decomposed into an elastic sliding velocity ${\bar{\text{v}}}^{e}$ and a plastic sliding velocity ${\bar{\text{v}}}^{p}$, i.e.

2.4 $$\bar{\text{v}}={\bar{\text{v}}}^{e}+{\bar{\text{v}}}^{p}.$$

The elastic sliding velocity is infinitesimal because it is induced by the elastic deformations of asperities on the contact surface; it is uniquely related to the rate of contact stress. On the other hand, the plastic sliding velocity is induced by the mutual slips between the surfaces of the main and the counter bodies and thus it is finite usually. Then, the direction of the plastic sliding velocity is given by the plastic potential function of the contact stress and thus depends on the contact stress but is independent of the rate of contact stress, as will be formulated in §2c(iii). Hence, the rate of contact stress is not determined uniquely by the sliding velocity, making it difficult to carry out the analysis if the elastic sliding velocity is not given. Therefore, we incorporate the elastic sliding velocity analogously to the elastic strain rate in ordinary elastoplastic constitutive equations.

Furthermore, ${\bar{\text{v}}}_{n}$ and ${\bar{\text{v}}}_{t}$ split additively into elastic and plastic parts as follows:

2.5 $${\bar{\text{v}}}_{n}={\bar{\text{v}}}_{n}^{e}+{\bar{\text{v}}}_{n}^{p},\;{\bar{\text{v}}}_{t}={\bar{\text{v}}}_{t}^{e}+{\bar{\text{v}}}_{t}^{p}$$

with

2.6 $${\bar{\text{v}}}^{e}={\bar{\text{v}}}_{n}^{e}+{\bar{\text{v}}}_{t}^{e}=-{\bar{v}}_{n}^{e}\text{n}+{\bar{v}}_{t}^{e}{\text{t}}_{v}^{e},\;{\bar{\text{v}}}^{p}={\bar{\text{v}}}_{n}^{p}+{\bar{\text{v}}}_{t}^{p}=-{\bar{v}}_{n}^{p}\text{n}+{\bar{v}}_{t}^{p}{\text{t}}_{v}^{p},$$

where

2.7 $${\bar{\text{v}}}_{n}^{e}=({\bar{\text{v}}}^{e}•\text{n})\text{n}=(\text{n}⊗\text{n}){\bar{\text{v}}}^{e}=-{\bar{v}}_{n}^{e}\text{n},\;{\bar{\text{v}}}_{t}^{e}={\bar{\text{v}}}^{e}-{\bar{\text{v}}}_{n}^{e}=(\text{I}-\text{n}⊗\text{n}){\bar{\text{v}}}^{e}={\bar{v}}_{t}^{e}{\text{t}}_{v}^{e}$$

and

2.8 $${\bar{\text{v}}}_{n}^{p}=({\bar{\text{v}}}^{p}•\text{n})\text{n}=(\text{n}⊗\text{n}){\bar{\text{v}}}^{p}=-{\bar{v}}_{n}^{p}\text{n},\;{\bar{\text{v}}}_{t}^{p}={\bar{\text{v}}}^{p}-{\bar{\text{v}}}_{n}^{p}=(\text{I}-\text{n}⊗\text{n}){\bar{\text{v}}}^{p}={\bar{v}}_{t}^{p}{\text{t}}_{v}^{p},$$

setting

2.9 $${\bar{v}}_{n}^{e}\equiv -\text{n}•{\bar{\text{v}}}_{n}^{e}=-\text{n}•{\bar{\text{v}}}^{e},\;{\bar{v}}_{t}^{e}=∥{\bar{\text{v}}}_{t}^{e}∥,\;{\text{t}}_{v}^{e}\equiv \frac{{\bar{\text{v}}}_{t}^{e}}{∥{\bar{\text{v}}}_{t}^{e}∥}\;\;(\text{n}•{\text{t}}_{v}^{e}=0,∥{\text{t}}_{v}^{e}∥=1)$$

and

2.10 $${\bar{v}}_{n}^{p}\equiv -\text{n}•{\bar{\text{v}}}_{n}^{p}=-\text{n}•{\bar{\text{v}}}^{p},\;{\bar{v}}_{t}^{p}=∥{\bar{\text{v}}}_{t}^{p}∥,\;{\text{t}}_{v}^{p}\equiv \frac{{\bar{\text{v}}}_{t}^{p}}{∥{\bar{\text{v}}}_{t}^{p}∥}(\text{n}•{\text{t}}_{v}^{p}=0,∥{\text{t}}_{v}^{p}∥=1).$$

The *contact stress (traction)* *vector* **f** acting on the main body orthogonally splits into the *normal traction* *vector* **f**_*n*_ and the *tangential traction vector* **f**_*t*_,

2.11 $$\text{f}={\text{f}}_{n}+{\text{f}}_{t}=-f_{n}\text{n}+f_{t}{\text{t}}_{f},$$

where

2.12 $${\text{f}}_{n}\equiv (\text{n}•\text{f})\text{n}=(\text{n}⊗\text{n})\text{f}=-f_{n}\text{n},\;{\text{f}}_{t}\equiv \text{f}-{\text{f}}_{n}=(\text{I}-\text{n}⊗\text{n})\text{f}=f_{t}{\text{t}}_{f}$$

and

2.13 $$f_{n}\equiv -\text{n}•\text{f},\;f_{t}=∥{\text{f}}_{t}∥,\;{\text{t}}_{f}\equiv \frac{{\text{f}}_{t}}{∥{\text{f}}_{t}∥}\;(\text{n}•{\text{t}}_{f}=0,∥{\text{t}}_{f}∥=1).$$

The minus sign is added to the definition of *f*_*n*_ such that friction is generated when the normal contact stress applied to the main body is compressive.

### (b) Elastic sliding velocity

Assuming that a solid does not split under moderate deformations, a hyperelastic constitutive relation possessing the elastic potential energy function is adopted for the elastic part of deformation in the exact finite strain theory [27]. By contrast, surface asperities on the main body adhere to different surface asperities on the counter body one after another during a sliding process and thus the hyperelastic relation would not hold in the friction phenomenon. Then, recalling the hypoelastic relation in terms of the Cauchy stress and the elastic strain rate (the symmetric part of the elastic velocity gradient) for the elastic deformation of solids [35], let the elastic sliding velocity be formulated as follows:

2.14 $${\bar{\text{v}}}^{e}={\text{C}}^{e-1}\overset{\;\circ }{\text{f}},\;\overset{\;\circ }{\text{f}}={\text{C}}^{e}{\bar{\text{v}}}^{e},$$

where (°) stands for the corotational rate [29]. The corotational time-derivative $\overset{\;\circ }{\text{f}}$ of contact stress in equation (2.11) is orthogonally decomposed into normal and tangential parts to the contact surface as

2.15 $$\overset{\;\circ }{\text{f}}={\overset{\;\circ }{\text{f}}}_{n}+{\overset{\;\circ }{\text{f}}}_{t},$$

where

2.16 $$\overset{\;\circ }{\text{f}}=\overset{\;•}{\text{f}}-\omega_{c}\text{f},\;{\overset{\;\circ }{\text{f}}}_{n}={\overset{\;•}{\text{f}}}_{n}-\omega_{c}{\text{f}}_{n},\;{\overset{\;\circ }{\text{f}}}_{t}={\overset{\;•}{\text{f}}}_{t}-\omega_{c}{\text{f}}_{t},$$

noting

2.17 $$\overset{\;\circ }{\text{f}}={\left({\text{f}}_{n}+{\text{f}}_{t}\right)}^{•}-\omega_{c}({\text{f}}_{n}+{\text{f}}_{t})={\overset{\;•}{\text{f}}}_{n}-\omega_{c}{\text{f}}_{n}+{\overset{\;•}{\text{f}}}_{t}-\omega_{c}{\text{f}}_{t}.$$

The second-order tensor ***ω***_*c*_ is the spin of the contact surface. The objectivity of the corotational contact stress rate $\overset{\;\circ }{\text{f}}$ in equation (2.16)_1_ can be confirmed by the fact that $\overset{\;\circ }{\text{f}}$ is formulated such that the influence of rigid-body rotation, ***ω***_*c*_**f**, is excluded from the material-time derivative $\overset{\;•}{\text{f}}$.

The second-order tensor **C**^e^ is the contact elastic modulus tensor given by

2.18 $${\text{C}}^{e}=\alpha_{n}\text{n}⊗\text{n}+\alpha_{t}(\text{I}-\text{n}⊗\text{n}),\;{\text{C}}^{e-1}=\frac{1}{\alpha_{n}}\text{n}⊗\text{n}+\frac{1}{\alpha_{t}}(\text{I}-\text{n}⊗\text{n}),$$

where *α*_*n*_ and *α*_*t*_ are the normal and tangential contact elastic constants, respectively, in the contact surface. These values are set large because the elastic sliding is caused by elastic deformations of the surface asperities. Actually, we may choose *α*_*n*_, *α*_*t*_=10^2^∼10^5^ GPa mm^−1^ for common metals as the penalty parameters. Equation (2.14) with equation (2.18) leads to

2.19 $${\bar{\text{v}}}^{e}=\frac{1}{\alpha_{n}}{\overset{\;\circ }{\text{f}}}_{n}+\frac{1}{\alpha_{t}}{\overset{\;\circ }{\text{f}}}_{t},\;\overset{\;\circ }{\text{f}}=\alpha_{n}{\bar{\text{v}}}_{n}^{e}+\alpha_{t}{\bar{\text{v}}}_{t}^{e}.$$

### (c) Formulation of plastic sliding velocity

In this section, the plastic sliding velocity is formulated on the basis of the concept of the subloading surface [20,29].

#### (i) Normal friction-yield and subloading-friction surfaces

Let the friction-yield surface be given by

2.20 f(f)=μ,

where *μ* is the hardening/softening function describing the expansion/contraction of the friction-yield surface. The friction-yield stress function *f*(**f**) for the Coulomb friction law is given by

2.21 $$f(\text{f})=\frac{f_{t}}{f_{n}},$$

for which *μ* specifies the coefficient of friction. Equation (2.21) will be adopted for actual calculations in later sections.

The abrupt transition from the elastic to the plastic sliding state is described if the interior of the friction-yield surface is assumed to be a purely elastic domain. However, the plastic sliding velocity is induced even by the rate of contact stress inside the friction-yield surface and it develops gradually as contact stress approaches the friction-yield surface, thereby exhibiting the smooth elastic–plastic transition. Then, to describe the plastic sliding velocity induced by the rate of contact stress inside the friction-yield surface, we incorporate the following postulate based on experimental facts [22,29].

*Fundamental postulate of unconventional elastoplasticity (subloading-surface concept)*. The contact stress approaches the friction-yield surface when the plastic sliding velocity is induced but it recedes from the friction-yield surface when only the elastic sliding velocity is induced.

**Figure 1.**
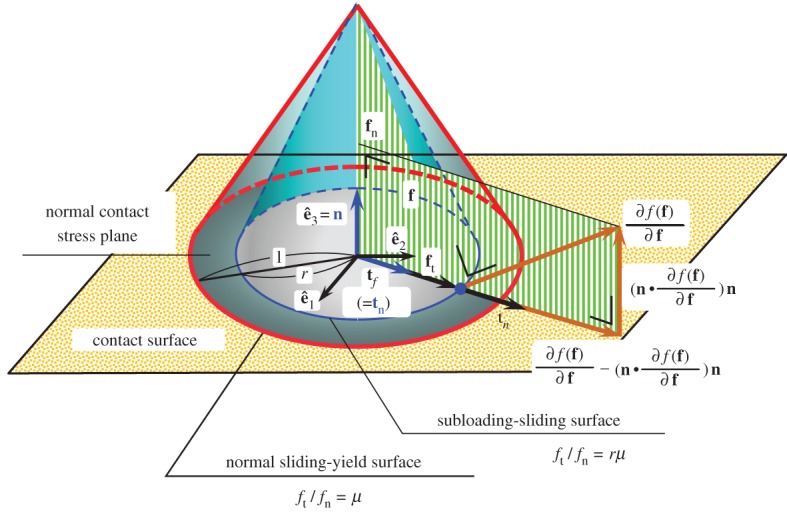
Coulomb-type normal friction-yield and subloading-friction surfaces. (Online version in colour.)

In this context, it is first required to incorporate the three-dimensional measure which describes the approaching degree of the contact stress to the friction-yield surface, renamed the *normal friction-yield surface*, in order to formulate the plastic sliding velocity based on the subloading-surface concept. Then, let the following *subloading-friction surface*, which always passes through the current contact stress and maintains a similar shape and orientation to the normal friction-yield surface, be introduced:

2.22 f(f)=rμ,

where *r* (0≤*r*≤1) is the ratio of the size of the subloading-friction surface to that of the normal friction-yield surface and is called the *normal friction-yield ratio*, playing the role of the measure of the approaching degree of contact stress to the normal friction-yield surface. For the Coulomb-type friction-yield surface, the normal friction-yield ratio is given by the ratio of the size of the cross-section of the subloading-friction surface to that of the normal friction-yield surface on the constant normal contact stress plane as shown in figure 1.

On the basis of the above-mentioned fundamental postulate of elastoplastic sliding, the rate of the normal friction-yield ratio *r* must satisfy the following conditions:

2.23 $$\overset{•}{r}\left\{ \begin{array}{ll} \to +\infty & \mathrm{for}\;r=0:\mathrm{quasi}\text{-}\mathrm{elastic}\;\mathrm{sliding}\;\mathrm{state}\\ >0 & \mathrm{for}\;0<r<1:\mathrm{sub}\text{-}\mathrm{friction}\text{-}\mathrm{yield}\;\mathrm{state}\\ =0 & \mathrm{for}\;r=1:\mathrm{normal}\;\mathrm{friction}\text{-}\mathrm{yield}\;\mathrm{state}\\ (<0 & \mathrm{for}\;r>1:\mathrm{over}\;\mathrm{normal}\;\mathrm{friction}\text{-}\mathrm{yield}\;\mathrm{state}) \end{array}\right.\;\mathrm{for}\;{\bar{\text{v}}}^{p}\neq \text{0}$$

and

2.24 $$\overset{•}{r}\left\{ \begin{array}{ll} =0 & \text{for}\;{\bar{\text{v}}}^{e}=\text{0}\\ <0 & \text{for}\;{\bar{\text{v}}}^{e}\neq \text{0} \end{array}\right.\;\mathrm{for}\;{\bar{\text{v}}}^{p}=\text{0}.$$

Taking equation (2.23) into account, let the evolution rule for the normal friction-yield ratio in the plastic sliding process be formulated as

2.25 $$\overset{•}{r}=\bar{U}(r)∥{\bar{\text{v}}}^{p}∥\;\mathrm{for}\;{\bar{\text{v}}}^{p}\neq \text{0},$$

where $\bar{U}(r)$ is a monotonically decreasing function of *r* satisfying the conditions

**Figure 2.**
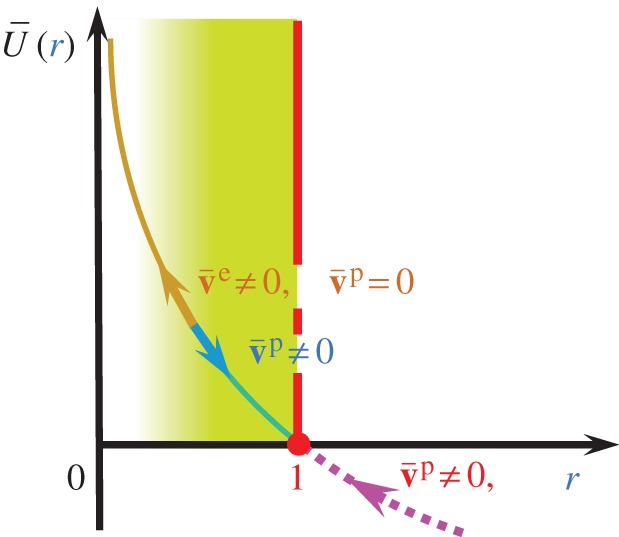
Function $\bar{U}(r)$ for the evolution rule of the normal friction-yield ratio *r*. (Online version in colour.)

2.26 $$\bar{U}(r)\left\{ \begin{array}{ll} \to +\infty & \text{for }r=0:\;\text{quasi-elastic sliding state}\\ >0 & \text{for }0<r<1:\text{ sub-friction-yield state}\\ =0 & \text{for }r=1:\text{ normal friction-yield state}\\ (<0 & \text{for}\;r>1:\text{ over normal friction-yield state}), \end{array}\right.$$

the general trend of which is illustrated in figure 2.

An explicit example of the function $\bar{U}(r)$ is

2.27 $$\bar{U}(r)=\tilde{u}\;\cot\left(\frac{\pi }{2}r\right),$$

where $\tilde{u}$ is a material constant. Equations (2.25) with equation (2.27) can be analytically integrated in the case of a monotonic sliding process as

2.28 $$r=\frac{2}{\pi }\cos^{-1}\left\{\cos\left(\frac{\pi }{2}r_{0}\right)\exp\left[-\frac{\pi }{2}\tilde{u}({\bar{u}}^{p}-{\bar{u}}_{0}^{p})\right]\right\},\;{\bar{u}}^{p}-{\bar{u}}_{0}^{p}=\frac{2}{\pi }\frac{1}{\tilde{u}}\;\ln\frac{\cos\left(\frac{\pi }{2}r_{0}\right)}{\cos\left(\frac{\pi }{2}r\right)},$$

where ${\bar{u}}^{p}=\int ∥{\bar{\text{v}}}^{p}∥\;dt$ is the accumulated plastic sliding displacement, and *r*_0_ and ${\bar{u}}_{0}^{p}$ are the initial values of *r* and ${\bar{u}}^{p}$, respectively. The analytical integration would be beneficial in implicit numerical calculations [22], while further work is required for the formulation of the implicit calculation method, i.e. a return-mapping projection based on the subloading-friction model. Here, it should be noted that numerical calculations can be performed with high efficiency by the forward-Euler method based on the subloading-friction model which possesses an automatic-controlling function to attract the contact stress to the normal friction-yield surface.

There exist the following functions other than the one in equation (2.27) which satisfy the conditions in equation (2.26), but unfortunately analytical integration does not exist for them:

2.29 $$\bar{U}(r)=-\tilde{u}\ln r,\;\bar{U}(r)=\tilde{u}\left(\frac{1}{r}-1\right).$$

**Figure 3.**
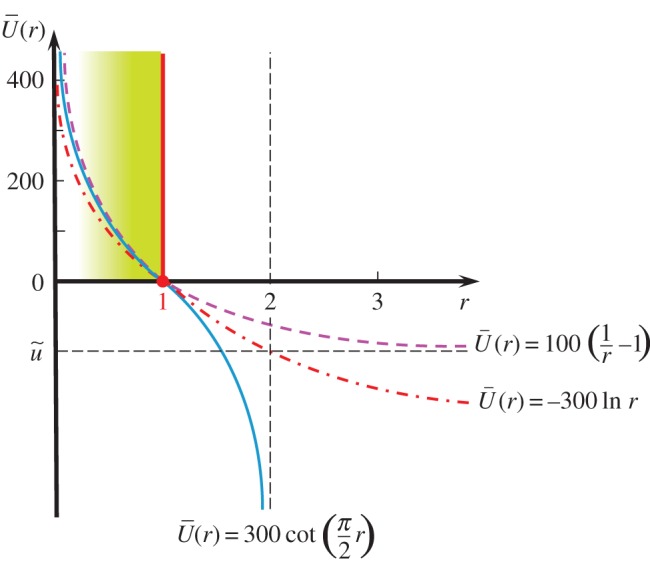
Three types of function $\bar{U}(r)$ in the evolution rule of the normal friction-yield ratio *r*. (Online version in colour.)

The three functional forms for $\bar{U}(r)$ in equations (2.27) and (2.29) are schematically depicted in figure 3, setting $\tilde{u}=100$ in equations (2.27) and (2.29)_2_ and $\tilde{u}=300$ in equation (2.29)_1_ so that the values of these functions coincide approximately to each other in the range *r*≤1. From figure 3, it follows that

2.30 $$\begin{array}{rl} & 100\;\cot\left(\frac{\pi }{2}r\right)≅-300\ln r≅100\left(\frac{1}{r}-1\right)\geq 0\;\mathrm{for}\;r\leq 1\\ \;\mathrm{and}\; & 100\cot\left(\frac{\pi }{2}r\right)<-300\ln r<100\left(\frac{1}{r}-1\right)<0\;\mathrm{for}\;r>1, \end{array}\}$$

noting

2.31 $$\begin{array}{r} r\to 2-0:\overset{•}{r}\to -\infty \;\mathrm{in}\;\tilde{u}\cot\left(\frac{\pi }{2}r\right);r\to \infty :\overset{•}{r}\to -\infty \;\mathrm{in}-\tilde{u}\ln r;r\to \infty :\overset{•}{r}\to -\tilde{u}\;\mathrm{in}\;\tilde{u}\left(\frac{1}{r}-1\right). \end{array}$$

The cotangent function in equation (2.27) will be adopted in subsequent analyses for the following reasons.

**Figure 4.**
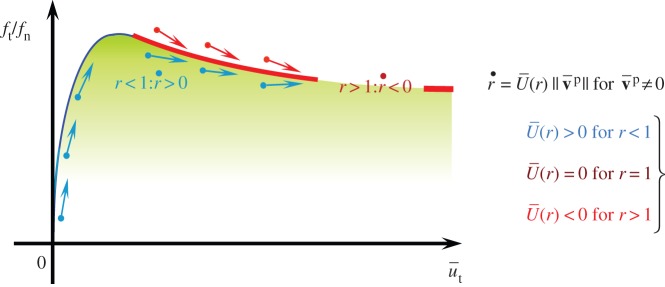
Contact stress controlling function in the subloading-friction model: contact stress is automatically attracted to the normal friction-yield surface in the plastic sliding process. (Online version in colour.)

(1) It possesses the largest negative value in the range *r*>1 among these three kinds of function $\bar{U}(r)$. Then, it provides the most intense controlling function to pull back the contact stress to the normal friction-yield surface when the stress jumps out from that surface in numerical calculations. Here, note that the evolution rule of the normal friction-yield ratio *r* is given by $\overset{•}{r}=\bar{U}(r)∥{\bar{\text{v}}}^{p}∥$ in equation (2.25), so that *r* becomes negative, i.e. $\overset{•}{r}<0$, and thus the contact stress is pulled back to the normal friction-yield surface if it goes over that surface, i.e. *r*>1, leading to $\bar{U}(r)<0$. This fact will be explained in detail in §2g with the illustration in figure 4.
(2) It can be analytically integrated as shown in equation (2.28) and it thereby provides an advantage in the implicit numerical calculation.

#### (ii) Evolution of the friction coefficient

The following characteristics for the variation of the friction coefficient are deduced from the hypothesis of adhesion in dry friction [36].

(1) The friction coefficient decreases with plastic sliding because the adhesion of surface asperities on the contact surface is broken by plastic sliding.
(2) The friction coefficient recovers with time because the adhesion of surface asperities is reconstructed as time elapses.

Then, the evolution rule for the friction coefficient is assumed as follows:

2.32 $$\overset{•}{\mu }=\kappa \underset{\mathrm{negative}}{\underbrace{\left(1-\frac{\mu }{\mu_{k}}\right)}}∥{\bar{\text{v}}}^{p}∥+\xi \underset{\mathrm{positive}}{\underbrace{\left(1-\frac{\mu }{\mu_{s}}\right)}},$$

where *μ*_s_ and *μ*_k_ (*μ*_s_≥*μ*≥*μ*_k_) are material constants designating the maximum and minimum values of *μ* for static friction and kinetic friction, respectively. *κ*(>0) is a material constant specifying the rate of decrease of *μ* in the plastic sliding process, and *ξ*(>0) is a material constant specifying the rate of recovery of *μ* as time elapses.

#### (iii) Plastic sliding velocity

The time differentiation of the subloading-friction surface in equation (2.22) reads

2.33 $$\frac{\partial f(\text{f})}{\partial \text{f}}•\overset{\;\circ }{\text{f}}=r\overset{•}{\mu }+\overset{•}{\text{r}}\mu .$$

Substituting equations (2.25) and (2.32) into equation (2.33), we have

2.34 $$\frac{\partial f(\text{f})}{\partial \text{f}}•\overset{\;\circ }{\text{f}}=r\left[\kappa \left(1-\frac{\mu }{\mu_{k}}\right)∥{\bar{\text{v}}}^{p}∥+\xi \left(1-\frac{\mu }{\mu_{s}}\right)\right]+\bar{U}(r)∥{\bar{\text{v}}}^{p}∥\mu .$$

Now, we assume that the plastic sliding velocity is induced in the direction of the projection of the outward-normal vector of the subloading-friction surface to the contact surface, which may be called the *tangent-associated flow rule*, i.e.

2.35 $${\bar{\text{v}}}^{p}=\overset{•}{\bar{\lambda }}{\text{t}}_{n}\left(\overset{•}{\bar{\lambda }}\geq 0\right)\left(∥{\bar{\text{v}}}^{p}∥=\overset{•}{\bar{\lambda }},\text{n}•{\bar{\text{v}}}^{p}=0\right)$$

with

2.36 $${\text{t}}_{n}\equiv \frac{\partial f(\text{f})/\partial \text{f}-(\text{n}•\partial f(\text{f})/\partial \text{f})\text{n}}{∥\partial f(\text{f})/\partial \text{f}-(\text{n}•\partial f(\text{f})/\partial \text{f})\text{n}∥}=\frac{(\text{I}-\text{n}⊗\text{n})\partial f(\text{f})/\partial \text{f}}{∥(\text{I}-\text{n}⊗\text{n})\partial f(\text{f})/\partial \text{f}∥}\left(∥{\text{t}}_{n}∥=1,\text{n}•{\text{t}}_{n}=0\right),$$

where $\overset{•}{\bar{\lambda }}$ and **t**_*n*_ are the magnitude and the direction, respectively, of the plastic sliding velocity. The vector ∂*f*(**f**)/∂**f**−[**n**•∂*f*(**f**)/∂**f**]**n** in equation (2.36) is the tangential part of the outward-normal vector ∂*f*(**f**)/∂**f** of the subloading-friction surface, and the vector **t**_*n*_ is its normalization. It follows from equations (2.8) and (2.35) with equation (2.36) that

2.37 $${\bar{\text{v}}}_{n}^{p}=\text{0},\;{\bar{\text{v}}}^{p}={\bar{\text{v}}}_{t}^{p}.$$

For the Coulomb friction-yield function described in equation (2.21), substituting

2.38 $$\frac{\partial f(\text{f})}{\partial \text{f}}=\frac{\partial }{\partial \text{f}}\left(\frac{f_{t}}{f_{n}}\right)=\frac{1}{f_{n}^{2}}\left(f_{n}\frac{\partial f_{t}}{\partial \text{f}}-f_{t}\frac{\partial f_{n}}{\partial \text{f}}\right)=\frac{1}{f_{n}^{2}}(\;f_{n}{\text{t}}_{f}-f_{t}(-\text{n}))=\frac{1}{f_{n}}\left({\text{t}}_{f}+\frac{f_{t}}{f_{n}}\text{n}\right)$$

into equation (2.36), it follows that the direction of the plastic sliding velocity coincides with the direction of the tangential contact stress vector defined in equation (2.13), i.e.

2.39 $${\text{t}}_{n}={\text{t}}_{f}.$$

Substituting equation (2.35) into equation (2.34), we have

2.40 $$\frac{\partial f(\text{f})}{\partial \text{f}}•\overset{\;\circ }{\text{f}}=\overset{•}{\bar{\lambda }}m^{p}+m^{c},$$

where

2.41 $$m^{p}\equiv \kappa \left(1-\frac{\mu }{\mu_{k}}\right)r+\bar{U}(r)\mu \;\mathrm{and}\;m^{c}\equiv \xi \left(1-\frac{\mu }{\mu_{s}}\right)r\;(\geq 0)$$

are relevant to the plastic and the creep sliding velocity, respectively.

It follows from equation (2.40) that

2.42 $$\overset{•}{\bar{\lambda }}=\frac{\partial f(\text{f})/\partial \text{f}•\overset{\;\circ }{\text{f}}-m^{c}}{m^{p}},\;{\bar{\text{v}}}^{p}=\frac{\partial f(\text{f})/\partial \text{f}•\overset{\;\circ }{\text{f}}-m^{c}}{m^{p}}{\text{t}}_{n}.$$

### (d) Relation between contact stress rate and sliding velocity

Substituting equations (2.14)_1_ and (2.42)_2_ into equation (2.4), we have

2.43 $$\bar{\text{v}}={\text{C}}^{e-1}\overset{\;\circ }{\text{f}}+\frac{\partial f(\text{f})/\partial \text{f}•\overset{\;\circ }{\text{f}}-m^{c}}{m^{p}}{\text{t}}_{n},$$

from which the magnitude of the plastic sliding velocity in terms of the sliding velocity, denoted $\overset{\;\;•}{\bar{\Lambda }}$, is derived as

2.44 $$\overset{\;\;•}{\bar{\Lambda }}=\frac{\partial f(\text{f})/\partial \text{f}•{\text{C}}^{e}\bar{\text{v}}-m^{c}}{m^{p}+\partial f(\text{f})/\partial \text{f}•{\text{C}}^{e}{\text{t}}_{n}}\left(=\frac{\partial f(\text{f})/\partial \text{f}•\overset{\circ }{\text{f}}-m^{c}}{m^{p}}\right).$$

The corotational rate of contact stress is obtained from equations (2.4), (2.14)_2_, (2.35) and (2.44) as follows:

2.45 $$\overset{\;\circ }{\text{f}}={\text{C}}^{e}\left(\bar{\text{v}}-\frac{\partial f(\text{f})/\partial \text{f}•{\text{C}}^{e}\bar{\text{v}}-m^{c}}{m^{p}+\partial f(\text{f})/\partial \text{f}•{\text{C}}^{e}{\text{t}}_{n}}{\text{t}}_{n}\right).$$

### (e) Loading criterion

The loading criterion for the plastic sliding velocity is given by

2.46 $$\begin{array}{rl} & {\bar{\text{v}}}^{p}\neq \text{0}:\overset{\;\;•}{\bar{\Lambda }}>0\\ & {\bar{\text{v}}}^{p}=\text{0}:\;\mathrm{otherwise}, \end{array}\}$$

or

2.47 $$\begin{array}{rl} & {\bar{\text{v}}}^{p}\neq \text{0}:\frac{\partial f(\text{f})}{\partial \text{f}}•{\text{C}}^{e}\bar{\text{v}}-m^{c}>0\\ & {\bar{\text{v}}}^{p}=\text{0}:\;\mathrm{otherwise}, \end{array}\}$$

noting $m^{p}+(\partial f(\text{f})/\partial \text{f})•{\text{C}}^{e}{\text{t}}_{n}>0$ in the denominator of the positive plastic multiplier in terms of the sliding velocity in equation (2.44). Here, note that the infinite plastic relaxation is generated so that the contact stress infinitely decreases if the denominator decreases to zero as known from equation (2.45).

### (f) Calculation of the normal friction-yield ratio

The normal friction-yield ratio *r* can be calculated using one of the following two methods.

(1) We calculate *r* using the following equation derived from the subloading-friction surface in equation (2.22) for both of the elastoplastic sliding and elastic sliding processes after the contact stress vector **f** and the hardening variable *μ* are updated:
  2.48 $$r=\frac{f(\text{f})}{\mu }.$$
  Needless to say, the hardening variable *μ* is calculated by the plastic sliding velocity in equation (2.42), including the plastic modulus *m*^*p*^, which depends on the evolution rule of the normal friction-yield ratio *r* in equation (2.25).
(2) We calculate *r* using method (1) for the elastic sliding process but calculate *r* by the time integration of equation (2.25) for the plastic sliding process. Actually, we adopt the function $\bar{U}(r)$ in equation (2.27) of the cotangent form as described in §2c(i). Here, it is beneficial to employ analytical time integration in equation (2.28) for the plastic loading process in implicit numerical calculations. However, in the numerical stress updating calculation by the forward-Euler method, we must use time integration based on the forward-Euler difference scheme for the rate-form evolution equation (2.25) to update the value of *r*. By this, the controlling function to attract the contact stress to the normal friction-yield surface works efficiently.

Method (2) would be superior to method (1) because the normal friction-yield ratio is calculated directly from the plastic sliding velocity.

### (g) Basic features of the subloading-friction model

The fundamental aspects of friction phenomena are described appropriately by the subloading-friction model as follows.

(1) The static friction as a peak and the decrease in the kinetic friction are described.
(2) The negative rate sensitivity is described because the friction coefficient decreases by plastic sliding and the recovery of the friction coefficient requires an elapse of time.
(3) The smooth transition from the elastic to the plastic sliding state is described, fulfilling the smoothness condition [37,38] because the plastic sliding velocity is not induced suddenly but induced gradually as the contact stress approaches the normal friction-yield surface so that a smooth contact stress versus sliding displacement curve is depicted, while the elastic sliding is usually quite small. Here, note that the smoothness of the transition is irrelevant to the elastic rigidity (magnitude of elastic sliding), as known from the existence of the smooth transition from the rigid to plastic transition.
(4) The yield judgement of whether the contact stress reaches the friction-yield surface is not required in the loading criterion in equation (2.46) or (2.47) because the plastic sliding velocity develops continuously as the contact stress approaches the normal friction-yield surface, excluding the assumption that the yield surface encloses a purely elastic domain.
(5) The contact stress is automatically attracted to the normal friction-yield surface in the plastic sliding process and it is pulled back to that surface when it jumps out from the normal friction-yield surface in numerical calculations by virtue of equation (2.25) with equation (2.26)_4_ leading to $\overset{•}{r}<0$ for *r*>1 as shown schematically in figure 4. It is known, by noting equation (2.30) with figures 3 and 4, that the cotangent function $\bar{U}(r)$ in equation (2.27) possesses the most intense controlling function to pull back the contact stress to the normal friction-yield surface among the three types of function $\bar{U}(r)$ in equations (2.27) and (2.29).

## 3. Generalized subloading-friction model: subloading-overstress friction model

In the subloading-friction model described in the preceding sections, the sliding velocity is postulated to consist of elastic and plastic sliding velocities. This leads to a negative rate sensitivity (i.e. a decrease in friction resistance with increasing sliding velocity) because the adhesion of surface asperities is broken more quickly at higher sliding velocities and the recovery of adhesion requires time. In what follows, we consider the generalization of the subloading-friction model to describe not only negative but also positive rate sensitivities by extending the notion of the overstress, which has been adopted in the description of viscoplastic deformation behaviour.

### (a) Formulation of the generalized subloading-friction model

First, we introduce the viscoplastic sliding velocity ${\bar{\text{v}}}^{vp}$ instead of the plastic sliding velocity ${\bar{\text{v}}}^{p}$ in equation (2.4), i.e.

3.1 $$\bar{\text{v}}={\bar{\text{v}}}^{e}+{\bar{\text{v}}}^{vp}.$$

Viscoplastic models describing rate-dependent plastic deformation are classified into either a creep model or an overstress model. A viscoplastic strain rate is always induced inappropriately depending on a stress level (e.g. [39]) or on the ratio of the magnitude of the stress to the yield stress (e.g. [40,41]) in creep models. By contrast, a viscoplastic strain rate is induced depending on the overstress from the yield surface in the overstress model [33,34]. In other words, although the creep model does not possess a loading criterion for the creep strain rate, the overstress model possesses the loading criterion for the viscoplastic strain rate. Here, it should be noted that a loading criterion for the viscoplastic strain rate as well as for the plastic strain rate in the ordinary elastoplastic constitutive equation is required for the realistic description of the rate-dependent elastoplastic deformation such that the viscoplastic strain rate is not induced when the stress is reduced from the yield surface. The subloading-friction model can then be generalized so as to describe both negative and positive rate sensitivities appropriately by incorporating the concept of overstress. First, let the viscoplastic sliding velocity be given as

3.2 $${\bar{\text{v}}}^{vp}=\frac{1}{\eta_{v}}{\left\langle \frac{f(\text{f})-\mu }{\mu }\right\rangle }^{n}{\text{t}}_{n}=\frac{1}{\eta_{v}}{\left\langle r-1\right\rangle }^{n}{\text{t}}_{n}.$$

Hence, by substituting equations (2.14) and (3.2) into equation (3.1), we have

3.3 $$\bar{\text{v}}={\text{C}}^{e-1}\overset{\;\circ }{\text{f}}+\frac{1}{\eta_{v}}{\left\langle r-1\right\rangle }^{n}{\text{t}}_{n},\;\overset{\;\circ }{\text{f}}={\text{C}}^{e}\bar{\text{v}}-\frac{1}{\eta_{v}}{\left\langle r-1\right\rangle }^{n}{\text{C}}^{e}{\text{t}}_{n},$$

where *η*_*v*_ and *n* are material constants. 〈 〉 denotes the Macaulay bracket, i.e. *s*<0:〈*s*〉=0 and *s*≥0:〈*s*〉=*s* (*s*: an arbitrary scalar). Note that the loading criterion for the viscoplastic sliding velocity is furnished by introducing the Macaulay bracket. The surface that passes through the current contact stress and that is similar to the normal friction-yield surface (similar on the constant normal contact stress plane for the Coulomb friction-yield surface) is called the *dynamic loading friction surface*. *r* is newly defined as the ratio of the size of the dynamic loading friction surface to that of the normal friction-yield surface and thus is called the *dynamic loading friction-yield ratio*. Here, note that *r*≤1 holds in the quasi-static sliding process but *r*>1 holds in the dynamic sliding process. Variations of internal variables are induced generally by irreversible (inelastic) sliding and hence the plastic sliding velocity ${\bar{\text{v}}}^{p}$ in the evolution rules of the internal variables in the elastoplastic sliding should be replaced by the viscoplastic sliding velocity ${\bar{\text{v}}}^{vp}$ in the evolution rules of the internal variables in the elasto-viscoplastic sliding. Then, from equation (2.32), the evolution rule for the coefficient of friction *μ* is generalized to

3.4 $$\overset{•}{\mu }=\kappa \underset{\mathrm{negative}}{\underbrace{\left(1-\frac{\mu }{\mu_{k}}\right)}}∥{\bar{\text{v}}}^{vp}∥+\xi \underset{\mathrm{positive}}{\underbrace{\left(1-\frac{\mu }{\mu_{s}}\right)}},$$

where *κ* and *ξ* are the material constants defined for equation (2.32).

Note here that equation (3.3) is rewritten in incremental forms,

3.5 $$d\bar{\text{u}}={\text{C}}^{e-1}d\text{f}+\frac{1}{\eta_{v}}{\left\langle r-1\right\rangle }^{n}{\text{t}}_{n}dt,\;d\text{f}={\text{C}}^{e}d\bar{\text{u}}-\frac{1}{\eta_{v}}{\left\langle r-1\right\rangle }^{n}{\text{C}}^{e}{\text{t}}_{n}\;dt,$$

where $d\bar{\text{u}}=\bar{\text{v}}dt$ is the increment of sliding displacement. In quasi-static sliding, equation (3.3) is reduced to

3.6 $$\text{0}≅\text{0}+\frac{1}{\eta_{v}}{\left\langle r-1\right\rangle }^{n}{\text{t}}_{n}\;dt,\;\text{0}≅\text{0}-\frac{1}{\eta_{v}}{\left\langle r-1\right\rangle }^{n}{\text{C}}^{e}{\text{t}}_{n}\;dt,$$

which leads to

3.7 r−1→0,i.e.  f(σ)−μ→0,

fulfilling the normal friction-yield condition without the overstress. In impact sliding, by contrast, equation (3.3) is reduced to

3.8 $$d\bar{\text{u}}≅{\text{C}}^{e-1}d\text{f}+\text{0},\;\text{i.e.}\;\bar{\text{v}}≅{\text{C}}^{e-1}\;\overset{\;\circ }{\text{f}},\;d\text{f}≅{\text{C}}^{e}\;d\bar{\text{u}}-\text{0},\;\text{i.e.}\;\overset{\;\circ }{\text{f}}≅{\text{C}}^{e}\bar{\text{v}},$$

which describe unrealistically the elastic sliding behaviour with an infinite friction strength, so that these equations are inapplicable to sliding phenomena at high sliding velocities. We then modify equation (3.3) as follows:

3.9 $$\bar{\text{v}}={\text{C}}^{e-1}\overset{\;\circ }{\text{f}}+\frac{1}{\eta_{v}}\frac{{\left\langle r-1\right\rangle }^{n}}{\hat{r}-r}{\text{t}}_{n},\;\overset{\;\circ }{\text{f}}={\text{C}}^{e}\bar{\text{v}}-\frac{1}{\eta_{v}}\frac{{\left\langle r-1\right\rangle }^{n}}{\hat{r}-r}{\text{C}}^{e}{\text{t}}_{n},$$

in which the viscoplastic sliding velocity becomes infinite as the dynamic loading friction-yield ratio *r* approaches the value of the material parameter $\hat{r}(>1)$, i.e. $r\to \hat{r}$. Then, we call $\hat{r}$ the *limit dynamic loading friction-yield ratio*.

The viscoplastic sliding velocity is induced suddenly at the moment that the contact stress reaches the normal friction-yield surface, i.e. when the dynamic loading friction-yield ratio becomes unity (*r*=1), so that a smooth elastic–viscoplastic transition cannot be described by equation (3.9). Then, we further modify these equations as follows:

3.10 $$\bar{\text{v}}={\text{C}}^{e-1}\overset{\;\circ }{\text{f}}+\frac{1}{\eta_{v}}\frac{{\left\langle r-r_{s}\right\rangle }^{n}}{\hat{r}-r}{\text{t}}_{n},\;\overset{\;\circ }{\text{f}}={\text{C}}^{e}\bar{\text{v}}-\frac{1}{\eta_{v}}\frac{{\left\langle r-r_{s}\right\rangle }^{n}}{\hat{r}-r}{\text{C}}^{e}{\text{t}}_{n}$$

by incorporating the variable *r*_s_ (0≤*r*_s_≤1), called the *subloading-friction-yield ratio*, which is calculated using equations (2.25) and (2.48) by replacing the plastic sliding velocity ${\bar{\text{v}}}^{p}$ with the viscoplastic sliding velocity ${\bar{\text{v}}}^{vp}$, i.e.

3.11 $${\overset{•}{r}}_{s}=\bar{U}(r_{s})∥{\bar{\text{v}}}^{vp}∥\;\mathrm{for}\;{\bar{\text{v}}}^{vp}\neq \text{0}$$

and

3.12 $$r_{s}=\frac{f(\text{f})}{\mu }\;\mathrm{for}\;{\bar{\text{v}}}^{\text{v}p}=\text{0}$$

with

3.13 $$\bar{U}(r_{s})=\tilde{u}\;\cot\left(\frac{\pi }{2}r_{s}\right).$$

Thus, the viscoplastic sliding velocity is induced by the overstress *f*(**f**)−*r*_s_*μ* from the subloading-friction surface

3.14 $$r-r_{s}=0,\;\text{i.e. }f(\text{f})=r_{s}\mu ,$$

so that a smooth elastic–viscoplastic transition is described.

Simulations reproducing test data are difficult when using equation (3.10) with a viscoplastic term in the power form but, as will be described in §5, high accuracy simulations can be performed by the use of the exponential function as

3.15 $$\bar{\text{v}}={\text{C}}^{e-1}\overset{\;\circ }{\text{f}}+\frac{1}{\eta_{v}}\;\frac{\left\langle e^{n(r-r_{s})}-1\right\rangle }{\hat{r}-r}{\text{t}}_{n},\;\overset{\;\circ }{\text{f}}={\text{C}}^{e}\bar{\text{v}}-\frac{1}{\eta_{v}}\;\frac{\left\langle e^{n(r-r_{s})}-1\right\rangle }{\hat{r}-r}{\text{C}}^{e}{\text{t}}_{n},$$

which will be used in subsequent sections. The generalized subloading-friction model proposed above is referred to as the *subloading-overstress friction model*.

**Figure 5.**
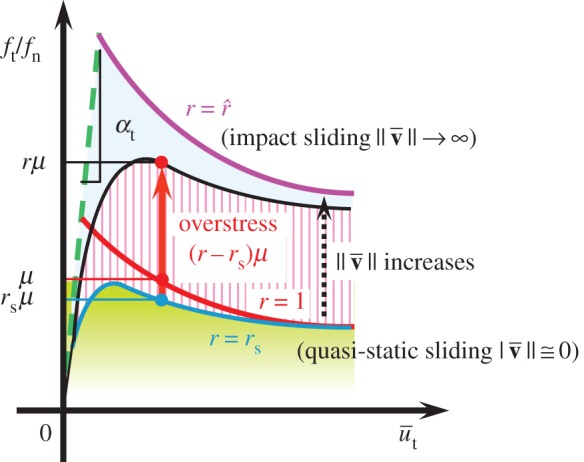
Response of the subloading-overstress friction model. (Online version in colour.)

The response of the subloading-overstress friction model for fluid friction is schematically shown in figure 5. The elastic sliding is depicted with exaggeration for a concise explanation, and the normalized overstress divided by the normal contact stress *f*_*n*_ is written simply as the term ‘overstress’ in this figure. The dynamic loading friction-yield ratio *r* coincides with the subloading-friction-yield ratio *r*_s_, i.e. *r*=*r*_s_ in quasi-static sliding and increases above *r*_s_ with increasing sliding velocity. However, *r* does not rise above $\hat{r}$, i.e. $r\leq \hat{r}$, while the equality $r=\hat{r}$ is realized only in impact sliding.

### (b) Interpretation of the subloading-overstress friction model

Equations (3.4) and (3.15) are described in incremental forms as

3.16 $$d\mu =\kappa \underset{\mathrm{negative}}{\underbrace{\left(1-\frac{\mu }{\mu_{k}}\right)}}∥d{\bar{\text{u}}}^{vp}∥+\xi \underset{\mathrm{positive}}{\underbrace{\left(1-\frac{\mu }{\mu_{s}}\right)}}dt$$

and

3.17 $$d\bar{\text{u}}={\text{C}}^{e-1}d\text{f}+\frac{1}{\eta_{v}}\frac{\left\langle e^{n(r-r_{s})}-1\right\rangle }{\hat{r}-r}{\text{t}}_{n}dt,\;d\text{f}={\text{C}}^{e}\;d\bar{\text{u}}-\frac{1}{\eta_{v}}\frac{\left\langle e^{n(r-r_{s})}-1\right\rangle }{\hat{r}-r}{\text{C}}^{e}{\text{t}}_{n}\;dt,$$

where $d{\bar{\text{u}}}^{vp}\equiv {\bar{\text{v}}}^{vp\;}dt$ is the viscoplastic sliding displacement.

The following properties are recognized from equations (3.16) and (3.17).

(1) In the quasi-static sliding process $\left(d\mu /dt≅0,∥d{\bar{\text{u}}}^{vp}∥/dt≅0,∥d\bar{\text{u}}∥/dt≅0,∥d\text{f}∥/dt≅0\right)$ in which the terms other than the second terms on the right-hand sides are negligible in equations (3.16) and (3.17), we have *μ*=*μ*_s_ from equation (3.16) and *r*=*r*_s_ from equation (3.17), resulting in *f*_*t*_/*f*_*n*_=*r*_s_*μ*_s_ due to equation (2.22) with equation (2.21). Therefore, the contact stress moves, satisfying the subloading-surface in the quasi-static sliding process, so that the original subloading-friction model is reproduced in that process. In other words, the subloading-overstress model is generalized so as to contain the subloading-friction model formulated for the dry friction in §2.
(2) In the fast sliding process $\left(d\mu /dt\to \infty ,∥d{\bar{\text{u}}}^{vp}∥/dt\to \infty ,∥d\bar{\text{u}}∥/dt\to \infty ,∥d\text{f}∥/dt\to \infty \right)$ for which the creep part of the second term on the right-hand side is negligible in equation (3.16), the friction coefficient *μ* decreases with the viscoplastic sliding displacement ${\bar{u}}^{vp}\equiv \int ∥{\bar{\text{v}}}^{vp}∥dt$ after reaching a peak.

**Figure 6.**
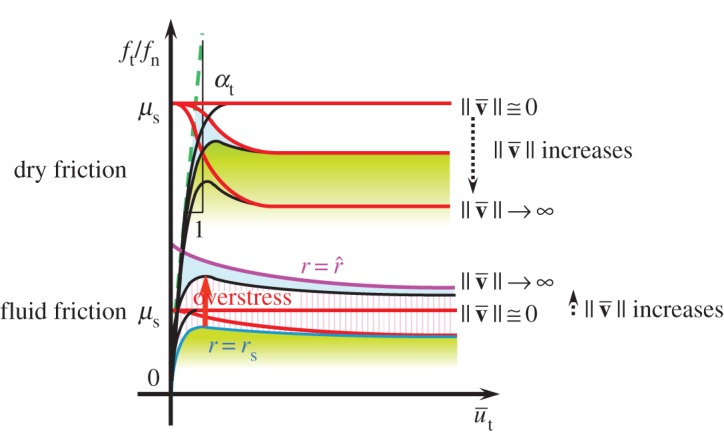
Comparison of responses in dry and fluid frictions at constant sliding velocities. (Online version in colour.)

Friction resistance varies with the destruction of the adhesion of surface asperities and the shearing of the viscous fluid lying between contact surfaces. The former is dominant in dry friction whereas the latter is dominant in fluid friction because the surfaces are in direct contact with each other in dry friction but are in indirect contact via the viscous medium in fluid friction. Then, adhesion of surface asperities is stronger in dry friction than in fluid friction. We thus infer the following differences between the responses of dry and fluid friction in the monotonic linear sliding process at constant sliding velocities (figure 6).

(3) The friction resistance is obviously greater in dry friction than in fluid friction, and the difference between a peak and a bottom contact stress ratio is larger in dry friction than in fluid friction.
(4) The sliding displacement in the transition from the peak to the bottom contact stress ratio is smaller in dry friction than in fluid friction because the contact is direct in the former but indirect via fluid in the latter.
(5) The friction coefficient *μ* decreases with sliding displacement, as described in (1). Then, dry friction exhibits a negative rate sensitivity because the contact stress ratio *f*_*t*_/*f*_*n*_ is given by the friction coefficient itself. On the other hand, fluid friction exhibits a positive rate sensitivity because the contact stress ratio is given by the friction coefficient plus the overstress, while the overstress increases with sliding velocity inducing a higher viscous resistance.
(6) The ratio *f*_*t*_/(*μ*_s_*f*_*n*_) approaches unity (i.e. *r*=1: normal sliding-yield state) in dry friction but becomes greater than unity (i.e. *r*>1: over normal sliding-yield state) in fluid friction.

In addition, note that it is not required to use equation (2.43) and/or equation (2.45) in the subloading-friction model even for the calculation of dry friction behaviour. In fact, the dry friction behaviour is generated in the quasi-static sliding process in the subloading-overstress friction model. Consequently, we need only use equation (3.15) with equations (3.4) and (3.11)–(3.13) in the subloading-overstress friction model for calculations of general sliding behaviour involving both dry and fluid frictions.

## 4. Numerical experiments

The basic mechanical properties of the subloading-overstress friction model formulated in the preceding section are examined below in numerical experiments using the constitutive equation (3.15) with equation (3.13) and adopting equation (2.21) for the Coulomb-type friction-yield stress function *f*(**f**).

In numerical simulations, the material constants in the subloading-friction model are chosen as follows:

$$\begin{array}{rl} & \text{Elastic sliding constant:}\;\alpha_{n}=\alpha_{t}=1000\;{\text{GPa mm}}^{-1}\\ & \text{Static friction coefficient:}\;\mu_{s}=0.097;\text{ Kinetic friction coefficient:}\;\mu_{k}=0.085\\ & \text{Sliding-softening constant:}\;\kappa =1.0\;{\mathrm{mm}}^{-1}\\ & \text{Creep-hardening constant:}\;\xi =0.005\;\min^{-1}\\ & \text{Normal friction-yield ratio evolution constant:}\;\tilde{u}=80\;{\mathrm{mm}}^{-1} \end{array}$$

Furthermore, the three material constants *η*_*v*_, *n* and $\hat{r}$ added in the subloading-overstress friction model are changed in the following five levels, fixing the other two material constants as *η*_*v*_=1000, *n*=16 and $\hat{r}=1.6$.

$$\begin{array}{rl} & \text{Viscoplastic coefficient:}\;\eta_{v}=10,100,1000,10\;000,100\;000\;{\min\;\mathrm{mm}}^{-1}\\ & \text{Viscoplastic power coefficient:}\;n=4,8,16,32,64\\ & \text{Limit dynamic loading friction-yield ratio:}\;\hat{r}=1.2,1.6,2.4,3.2,6.4 \end{array}$$

**Figure 7.**
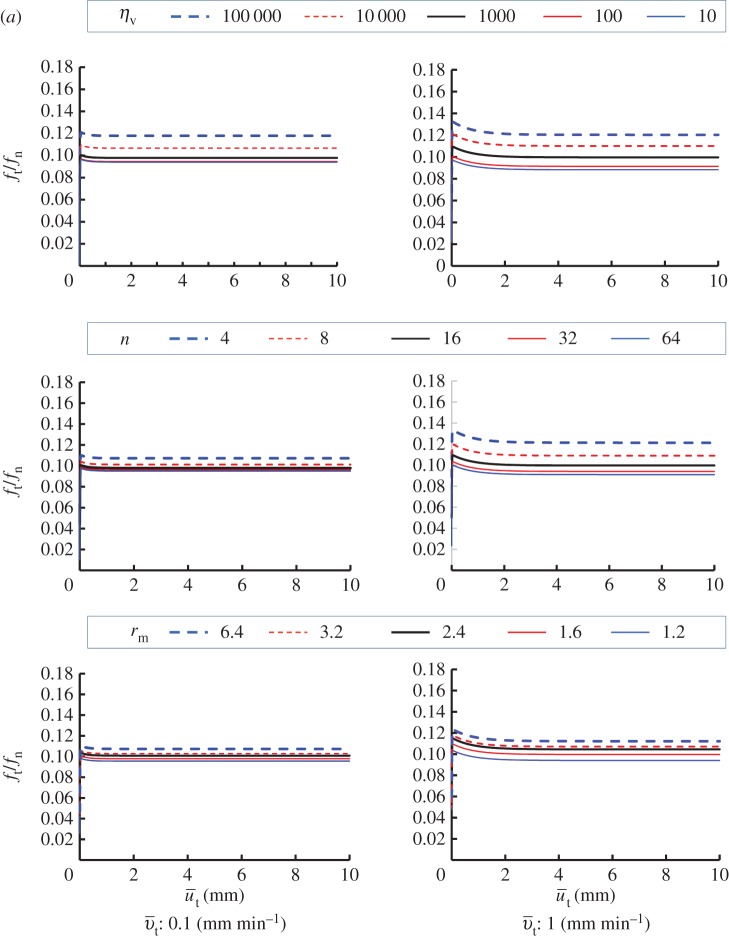

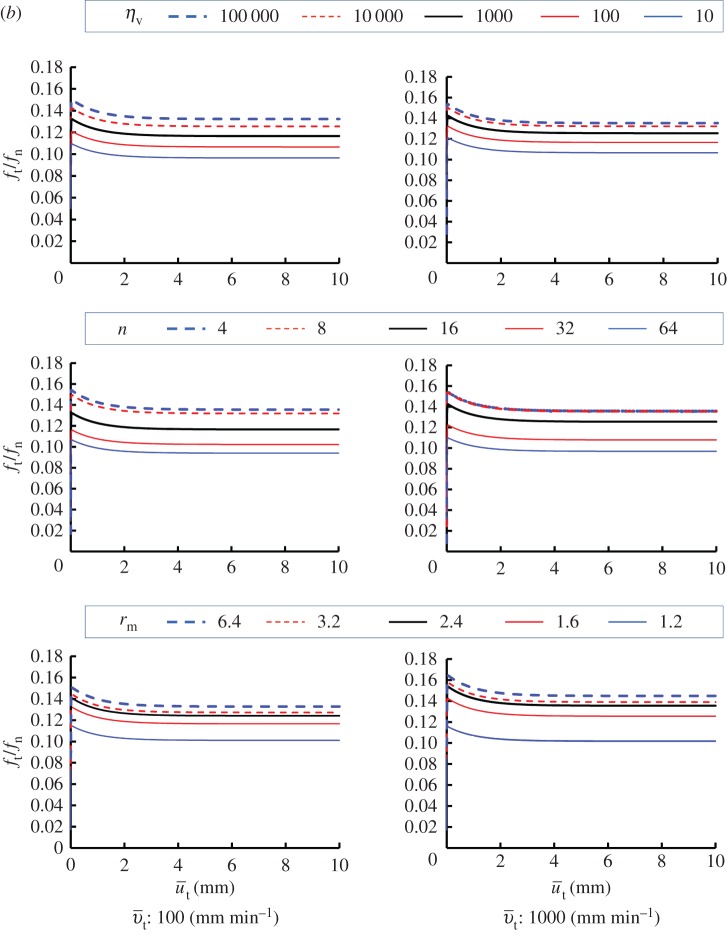
(*a*,*b*) Influence of material parameters in the subloading-overstress friction model.

**Figure 8.**
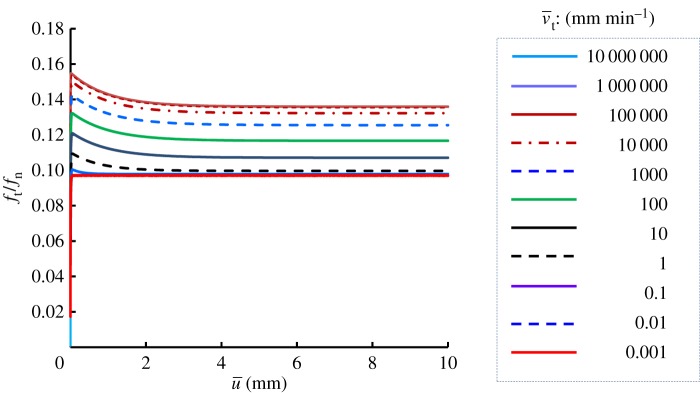
Influence of sliding velocity.

**Figure 9.**
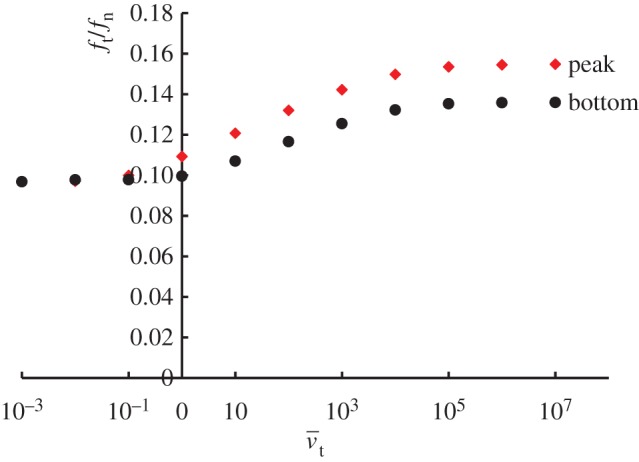
Influence of sliding velocity on the contact stress ratio. (Online version in colour.)

The sliding velocity is set to be 0.1, 1, 100 and 1000 mm min^−1^. The calculated results are shown in figure 7. The contact stress ratio *f*_*t*_/*f*_*n*_ is larger for higher sliding velocities, exhibiting positive rate sensitivity. Higher contact stress ratios are predicted for larger values of *η*_*v*_ and $\hat{r}$, and for lower values of *n*. Curves of the contact stress ratio versus sliding displacement for various sliding velocities are shown in figure 8, indicating positive rate sensitivity, where the material constants are chosen as *η*_*v*_=1000, *n*=16 and $\hat{r}=1.6$. The effect of the sliding velocity on the peak (maximum) and bottom (minimum) contact stress ratios in the contact stress ratio versus sliding displacement curves, which are read from figure 8, are plotted in figure 9. Here, the peak and bottom contact stress ratios increase with sliding velocity in a certain velocity range, whereas they converge to certain values outside this range.

## 5. Comparison with test data

**Figure 10.**
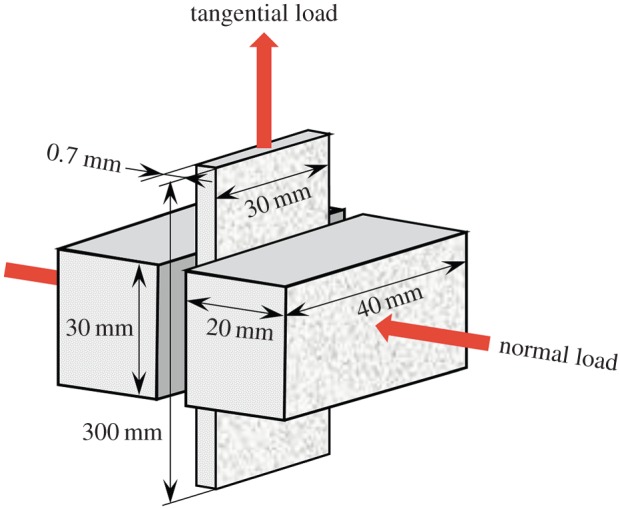
Illustration of the friction test apparatus. (Online version in colour.)

To examine the validity of the generalized subloading-overstress model, a lubricated friction test was performed. A schematic diagram of the test apparatus is shown in figure 10. The test plate, which is placed between the tools, is subjected to the constant normal load (5 kN) and is pulled up at constant velocity. The pulling force is measured by the load cell (maximum load: 100 kN) attached to the upper part of the test plate. The test plate is made of galvannealed steel sheet with a friction area of width 30 mm, height 300 mm and thickness 0.7 mm. The tool steel SKD-11 of width 40 mm, height 30 mm and thickness 20 mm is used for the tools which grasp the test plate. Therefore, the friction contact area for the normal contact stress is 900 mm^2^ and therefore the normal contact stress is 5.56 MPa, whereas the friction contact area for the tangential contact stress is 1800 mm^2^. The friction surfaces were polished and coated with anti-rust oil prior to the tests. The pulling-up velocity of the test plate is set at five levels: 1, 10, 50, 100, 200 mm min^−1^. The friction test was adopted supposing the press forming of thin sheet metal in the metal forming process.

**Figure 11.**
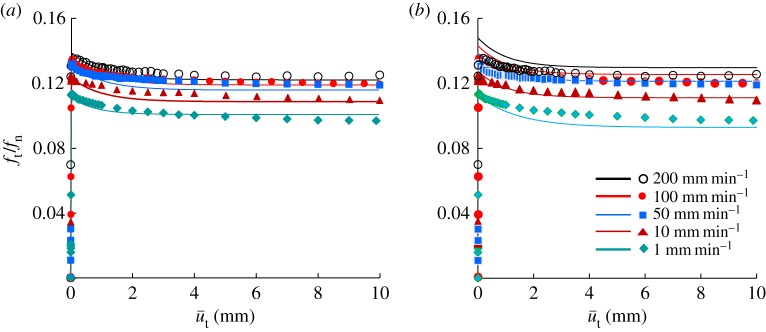
Comparison with test data for various levels of sliding velocity. (*a*) Equation (3.15) with an exponential function and (*b*) equation (3.10) with a power function. (Online version in colour.)

The measured relationship between the contact stress ratio *f*_t_/*f*_n_ and the tangential sliding displacement ${\bar{u}}_{t}$ is shown in figure 11*a*. The contact stress ratio first peaks and then gradually falls to a stationary value, exhibiting a positive rate sensitivity, i.e. larger contact stress ratios at higher sliding velocities.

The simulation of the above-mentioned test result using equation (3.15) with equations (2.21) and (3.13) is shown by the solid lines in figure 11*a*, using the following values for the material constants:

$$\begin{array}{rl} & \alpha_{n}=\alpha_{t}=1000\;{\text{GPa mm}}^{-1}\\ & \mu_{s}=0.097,\mu_{k}=0.085\\ & \kappa =0.2\;{\mathrm{mm}}^{-1},\xi =0.009\;\min^{-1}\\ & \tilde{u}=80\;{\mathrm{mm}}^{-1}\\ & \eta_{v}=950\;\min\;{\mathrm{mm}}^{-1},n=16,\hat{r}=1.8 \end{array}$$

The simulation of the test result using equation (3.10) with the power function is shown by the solid lines in figure 11*b*, using the following values for material constants. These material constants are chosen so as to simulate the test result as closely as possible. However, it is impossible to restrict the range from the highest and the lowest curves to the range in the test curves. Equation (3.15) with the exponential function would be much more appropriate than equation (3.10) with the power function for the prediction of real friction behaviour. However, clarification of the definite physical background should be continued for the future:

$$\begin{array}{rl} & \alpha_{n}=\alpha_{t}=1000\;{\text{GPa mm}}^{-1}\\ & \mu_{s}=0.097,\mu_{k}=0.085\\ & \kappa =1.0\;{\mathrm{mm}}^{-1},\xi =0.005\;\min^{-1}\\ & \tilde{u}=80\;{\mathrm{mm}}^{-1}\\ & \eta_{v}=950\;\min\;{\mathrm{mm}}^{-1},n=16,\hat{r}=1.8 \end{array}$$

**Figure 12.**
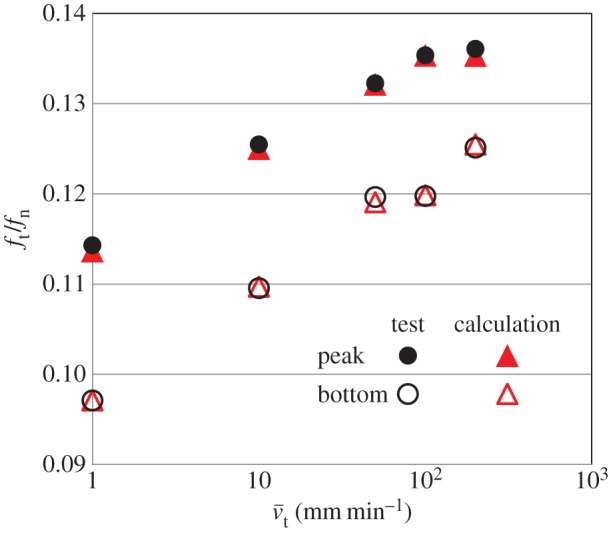
Comparison of calculation and test results on the influence of the sliding velocity on the contact stress ratio. (Online version in colour.)

Based on figure 11*a*, a comparison of the calculated and test results for the influence of the sliding velocity on the peak (maximum) and bottom (minimum) values in the contact stress ratio versus sliding displacement curves is shown in figure 12, where the close coincidence can be observed.

## 6. Concluding remarks

A generalized subloading-friction model, i.e. the subloading-overstress friction model, was formulated and the validity was verified in numerical experiments and by comparison with test data. The model is able to describe the following basic behaviours of friction phenomena.

(1) Peak and subsequent minimum friction resistance at a constant sliding velocity and the recovery of friction resistance as sliding stops or the sliding velocity decreases.
(2) Both negative and positive rate sensitivities in the unified form, while only the former has been described by the subloading-friction model [30,31].
(3) Sliding behaviour at general sliding velocity ranging from quasi-static to impact sliding.

The generalized subloading-friction model is formulated using the general friction-yield surface but the Coulomb friction-yield surface was adopted in the explicit analyses to explain the fundamental behaviour of the model concisely. The subloading-overstress friction model can be readily extended to describe the effect of the normal contact stress and the anisotropy on the friction resistance by incorporating the more general friction-yield surface which is the nonlinear function of the normal contact stress [30,31] and takes account of the orthotropic and rotational anisotropies [42,43].

Crystal plasticity is widely analysed by inappropriate methods using a creep model [40,41,44]. The subloading-overstress friction model, regarding the tangential contact stress and the tangential contact yield stress as the resolved shear stress and the critical shear stress, respectively, provides a rigorous analysis with high efficiency for rate-dependent crystal plasticity in addition to rate-independent crystal plasticity [28,29]. Details of the results of the calculation of crystal plasticity are forthcoming. Furthermore, the subloading-friction model can be applied to the prediction of continental slip-type earthquakes to prevent earthquake disasters in the near future.

## References

[1] Dieterich JH 1978 Time-dependent friction and the mechanism of stick-slip. *Pure Appl. Geophys.* 116, 790–806. (doi:10.1007/BF00876539)

[2] Ruina AL 1983 Slip instability and state variable friction laws. *J. Geophys. Res.* 88, 10 359–10 370. (doi:10.1029/JB088iB12p10359)

[3] Rice JR, Ruina AL 1983 Stability of steady frictional slipping. *J. Appl. Mech. (ASME)* 50, 343–349. (doi:10.1115/1.3167042)

[4] Rice JR, Lapusta N, Ranjith K 2001 Rate and state dependent friction and the stability of sliding between elastically deformable solids. *J. Mech. Phys. Solids* 49, 1865–1898. (doi:10.1016/S0022-5096(01)00042-4)

[5] Dieterich JH 1979 Modeling of rock friction 1. Experimental results and constitutive equations. *J. Geophys. Res.* 84, 2161–2168. (doi:10.1029/JB084iB05p02161)

[6] Ruina AL 1980 Friction laws and instabilities: quasistatic analysis of some dry frictional behavior. PhD thesis, Brown University, Providence, RI.

[7] Dieterich JH, Kilgore BD 1994 Direct observation of frictional contacts: new insights for state dependent properties. *Pure Appl. Geophys.* 143, 283–302. (doi:10.1007/BF00874332)

[8] Seguchi Y, Shindo A, Tomita Y, Sunohara M 1974 Sliding rule of friction in plastic forming of metal. *Compt. Meth. Nonlinear Mech.*, 683–692.

[9] Fredriksson B 1976 Finite element solution of surface nonlinearities in structural mechanics with special emphasis to contact and fracture mechanics problems. *Comput. Struct.* 6, 281–290. (doi:10.1016/0045-7949(76)90003-1)

[10] Oden JT, Pires EB 1983 Algorithms and numerical results for finite element approximations of contact problems with non-classical friction laws. *Comput. Struct.* 19, 137–147. (doi:10.1016/0045-7949(84)90212-8)

[11] Kikuchi N, Oden JT 1988 *Contact problem in elasticity: a study of variational inequalities and finite element methods*. Philadelphia, PA: SIAM.

[12] Wriggers P, Vu Van T, Stein E 1990 Finite element formulation of large deformation impact-contact problems with friction. *Comput. Struct.* 37, 319–331. (doi:10.1016/0045-7949(90)90324-U)

[13] Perić D, Owen DRJ 1992 Computational model for 3-D contact problems with friction based on the penalty method. *Int. J. Numer. Meth. Eng.* 35, 1289–1309. (doi:10.1002/nme.1620350609)

[14] Anand L 1993 A constitutive model for interface friction. *Comput. Mech.* 12, 197–213. (doi:10.1007/BF00369962)

[15] Mroz Z, Stupkiewicz S 1994 An anisotropic friction and wear model. *Int. J. Solids Struct.* 31, 1113–1131. (doi:10.1016/0020-7683(94)90167-8)

[16] Wriggers P 2003 *Computational contact mechanics*. Hoboken, NJ: John Wiley & Sons, Ltd.

[17] Oden JT, Martines JAC 1986 Models and computational methods for dynamic friction phenomena. *Comput. Meth. Appl. Mech. Eng.* 52, 527–634. (doi:10.1016/0045-7825(85)90009-X)

[18] Gearing BP, Moon HS, Anand L 2001 A plasticity model for interface friction: application to sheet metal forming. *Int. J. Plasticity* 17, 237–271. (doi:10.1016/S0749-6419(00)00034-6)

[19] Drucker DC 1988 Conventional and unconventional plastic response and representation. *Appl. Mech. Rev. (ASME)* 41, 151–167. (doi:10.1115/1.3151888)

[20] Hashiguchi K 1980 Constitutive equations of elastoplastic materials with elastic-plastic transition. *J. Appl. Mech. (ASME)* 47, 266–272. (doi:10.1115/1.3153653)

[21] Hashiguchi K 1989 Subloading surface model in unconventional plasticity. *Int. J. Solids Struct.* 25, 917–945. (doi:10.1016/0020-7683(89)90038-3)

[22] Hashiguchi K 2013 *Elastoplasticity theory*. Lecture Notes in Appl. Compt. Mech, 2nd edn Heidelberg, Germany: Springer.

[23] Hashiguchi K, Ueno M, Ozaki T 2012 Elastoplastic model of metals with smooth elastic-plastic transition. *Acta Mech.* 223, 985–1013. (doi:10.1007/s00707-012-0615-2)

[24] Hashiguchi K 2015 Subloading-damage constitutive equation. *Proc. Compt. Eng. Conf. Japan* 20, D-2-4.

[25] Hashiguchi K, Okamura K 2014 Subloading phase-transformation model. In *Proc. 27th JSME Compt. Mech. Div. Conf., Yokohama, Japan*, pp. OS17–1707. Tokyo, Japan: JSME.

[26] Higuchi R, Okamura K, Ohta F, Hashiguchi K 2014 Extension of subloading surface model for accurate prediction of elastoplastic deformation behavior of metals with cyclic softening. *Trans. Japan. Soc. Mech. Eng., Ser. A* 80, SMM0082. (doi:10.1299/transjsme.2014smm0082) (in Japanese).

[27] Hashiguchi K, Yamakawa Y 2012 *Introduction to finite strain theory for continuum elasto-plasticity*. Wiley Series in Computational Mechanics Chichester, UK: John Wiley & Sons Ltd.

[28] Hashiguchi K 2015 Exact formulation of subloading surface model: unified constitutive law for irreversible mechanical phenomena in solids. *Arch. Comput. Meth. Eng.* 22, 1–31. (doi:10.1007/s11831-015-9148-x)

[29] Hashiguchi K 2013 General description of elastoplastic deformation/sliding phenomena of solids in high accuracy and numerical efficiency: subloading surface concept. *Arch. Comput. Meth. Eng.* 20, 361–417. (doi:10.1007/s11831-013-9089-1)

[30] Hashiguchi K, Ozaki S, Okayasu T 2005 Unconventional friction theory based on the subloading surface concept. *Int. J. Solids Struct.* 42, 1705–1727. (doi:10.1016/j.ijsolstr.2004.08.006)

[31] Hashiguchi K, Ozaki S 2008 Constitutive equation for friction with transition from static to kinetic friction and recovery of static friction. *Int. J. Plasticity* 24, 2102–2124. (doi:10.1016/j.ijplas.2008.03.004)

[32] Ozaki S, Hashiguchi K 2010 Numerical analysis of stick-slip instability by a rate-dependent elastoplastic formulation for friction. *Tribol. Int.* 43, 2120–2133. (doi:10.1016/j.triboint.2010.06.007)

[33] Bingham EC 1922 *Fluidity and plasticity*. New York, NY: McGraw-Hill.

[34] Perzyna P 1963 The constitutive equations for rate sensitive plastic materials. *Q. Appl. Math.* 20, 321–332.

[35] Truesdell C 1955 Hypo-elasticity. *J. Rational Mech. Anal.* 4, 83–133.

[36] Bowden FP, Tabor D 1958 *The friction and lubrication of solids*. Oxford, UK: Clarendon Press.

[37] Hashiguchi K 1997 The extended flow rule in plasticity. *Int. J. Plasticity* 13, 37–58. (doi:10.1016/S0749-6419(96)00052-6)

[38] Hashiguchi K 2000 Fundamentals in constitutive equation: continuity and smoothness conditions and loading criterion. *Soils Foundations* 40 (3), 155–161. (doi:10.3208/sandf.40.4_155)

[39] Norton FH 1929 *Creep theory at high temperature*. New York, NY: McGraw-Hill.

[40] Hutchinson JW 1976 Bounds and self-consistent estimates for creep of polycrystalline materials. *Proc. R. Soc. Lond. A* 348, 101–127. (doi:10.1098/rspa.1976.0027)

[41] Pan J, Rice JR 1983 Rate sensitivity of plastic flow and implications for yield-surface vertices. *Int. J. Solids Struct.* 19, 973–987. (doi:10.1016/0020-7683(83)90023-9)

[42] Hashiguchi K 2007 Anisotropic constitutive equation of friction with rotational hardening. In *Proc. 13th Int. Symp. Plasticity and its Current Appl., Girdwood, Alaska, 2–6 June 2007*, pp. 34–36. New York, NY: Elsevier.

[43] Ozaki S, Hikida K, Hashiguchi K 2012 Elastoplastic formulation for friction with orthotropic anisotropy and rotational hardening. *Int. J. Solids Struct.* 49, 648–657. (doi:10.1016/j.ijsolstr.2011.11.010)

[44] Peirce D, Asaro RJ, Needleman A 1983 Overview 32: material rate dependence and localized deformation in crystal solids. *Act. Metall.* 31, 1951–1976. (doi:10.1016/0001-6160(83)90014-7)

